# Male histone deacetylase 6 (HDAC6) knockout mice have enhanced ventilatory responses to hypoxic challenge

**DOI:** 10.21203/rs.3.rs-3005686/v1

**Published:** 2023-06-13

**Authors:** Paulina M. Getsy, Gregory A. Coffee, Thomas J. Kelley, Stephen J. Lewis

**Affiliations:** Case Western Reserve University

**Keywords:** C57BL/6 mice, breathing, hypoxic gas challenge, Histone deacetylase 6 (HDAC 6) knockout mice

## Abstract

Histone deacetylase 6 (HDAC6) is a class II histone deacetylase that is predominantly localized in the cytoplasm of cells. HDAC6 associates with microtubules, regulating acetylation of tubulin and other proteins. The possibility that HDAC6 participates in hypoxic signaling is supported by evidence that (1) hypoxic gas challenges cause microtubule depolymerization, (2) expression of hypoxia inducible factor alpha (HIF)-1α is regulated by microtubule alterations in response to hypoxia, and (3) inhibition of HDAC6 prevents HIF-1α expression and protects tissue from hypoxic/ischemic insults. The aim of this study was to address whether the absence of HDAC6 alters ventilatory responses during and/or after hypoxic gas challenges (10% O_2_, 90% N_2_ for 15 min) in adult male wild-type (WT) C57BL/6 mice and HDAC6 knockout (KO) mice. Key findings were that (1) baseline values for frequency of breathing, tidal volume, inspiratory and expiratory times and end expiratory pause were different between KO mice and WT mice, (2) ventilatory responses during hypoxic challenge were more robust in KO mice than WT mice for parameters including frequency of breathing, minute ventilation, inspiratory and expiratory durations, peak inspiratory and expiratory flows, inspiratory and expiratory drives, and (3) responses upon return to room-air were markedly different in KO mice than WT mice for frequency of breathing, minute ventilation, inspiratory and expiratory durations, end expiratory (but not end inspiratory) pauses, peak inspiratory and expiratory flows, and inspiratory or expiratory drives. These data suggest that HDAC6 may have a fundamentally important role in regulating the neural responses to hypoxia.

## Introduction

Histone deacetylase 6 (HDAC6) is a class II histone deacetylase that exists predominantly within the cytosolic compartment of cells where it associates with microtubules to regulate the acetylation of tubulin and other cytosolic/intracellular protein targets.^1–6^ Numerous studies have demonstrated that pharmacological inhibition of HDAC6 improves neuronal function in multiple disease states.^1–3,7–12^ For example, the inhibition of HDAC6 improves microtubule-mediated transport in neurons in Huntington’s disease directly by increasing tubulin acetylation.^13^ The peripheral nerve disease, Charcot-Marie-Tooth, is characterized by reduced tubulin acetylation.^14^ HDAC6 inhibitors improve neuronal transmission and alleviate phenotypes in a mouse model of this disease.^14,15^ In addition, HDAC6 inhibitors have been assessed in vascular dementia (e.g., Alzheimer’s) diseases and Parkinson’s disease based on their ability to improve neuronal function via tubulin acetylation.^16–19^

Clear relationships between hypoxia and microtubule regulation have been demonstrated in cardiomyocyte preparations.^20,21^ For example, Dang et al^20^ demonstrated that hypoxic challenge leads to microtubule depolymerization. These findings are consistent with those from Teng et al^21^ who demonstrated that hypoxia inducible factor-1α (HIF-1α) expression is regulated by microtubule alterations in response to hypoxic challenge. Stable microtubules are preferentially acetylated, suggesting that HDAC6 inhibition should protect against damage induced by hypoxic challenge. Multiple studies have demonstrated that HDAC6 inhibition prevents HIF-1α expression and protects tissue from hypoxic/ischemia challenges.^22–25^ Taken together, it is evident that pharmacological inhibition of HDAC6 protects against hypoxic challenge-induced tissue damage and also improves central and peripheral neuronal function in a variety of disease states.^1–25^

Ventilatory responses to hypoxia are dependent on carotid body sensing and neuronal (chemoreceptor afferent) signal propagation to the commissural nucleus tractus solitarius in the brainstem.^26–29^ At present, there is no information as to (1) whether HDAC6 exists in primary glomus (hypoxia-sensing) cells within the carotid bodies or in key brain structures such as the commissural nucleus tractus solitarius that receive and process chemoreceptor afferent input. The evidence that HDAC6 inhibition prevents HIF-1α expression and protects tissue from hypoxic and/or ischemic damage^30–33^ is consistent with a role for HDAC6 in carotid body function since there is extensive evidence that HIF-1α plays many roles in hypoxic signaling in primary carotid body glomus cells.^34–39^

To our knowledge, there are no studies that have directly addressed whether or not HDAC6 has a role in signaling processes involved in expression of the ventilatory responses that occur upon exposure to hypoxic challenges. Accordingly, the primary objective of this study was to compare the ventilatory responses elicited by a hypoxic gas challenge (10% O_2_, 90% N_2_) in adult male wild-type (WT) C57BL/6 mice and HDAC6 knock-out (KO) mice using whole-body plethysmography.^40–46^ The data from these experiments clearly demonstrate that HDAC6 has a fundamentally important role in regulating the neural responses that drive the ventilatory responses to hypoxic challenge.

## Experimental Procedures

### Mice:

Adult wild type C57BL/6 mice were obtained from Jackson Laboratories (Bar Harbor, Maine). Adult male and female HDAC6 KO mice were generously provided by Dr. Tso-Pang Yao (Duke University). Breeding pairs of these mice provided the adult male HDAC6 KO mice used in this study. All studies described were carried out in accordance with the National Institutes of Health Guide for the Care and Use of Laboratory Animals (NIH Publication No. 80 – 23) revised in 1996 and in strict compliance with the ARRIVE (*Animal Research: Reporting of In Vivo Experiments*) guidelines (https://arriveguidelines.org). The protocols were approved by the Animal Care and Use Committee of Case Western Reserve University.

### Whole-body plethysmography

Ventilatory parameters in freely-moving mice were recorded by whole body plethysmography (PLY3223; *Data Sciences International*, St. Paul, MN) as described previously.^40–46^ The parameters (see **Supplemental Table 1, Supplemental Fig. 1**) were frequency of breathing (**Freq**); tidal volume (**TV**, volume of inspired air per breath), minute ventilation (Freq × TV, total volume of air inspired/min); inspiratory time (**Ti**, duration of inspiration); expiratory time (**Te**, duration of expiration); expiratory/inspiratory time (**Te/Ti**, expiratory quotient); end inspiratory pause (**EIP**, pause between end of inspiration and start of expiration); end expiratory pause (**EEP**, pause between end of expiration and start of inspiration); peak inspiratory flow (**PIF**); peak expiratory flow (**PEF**); airflow at 50% expired TV (**EF**_**50**_), relaxation time (**RT**, time to exhale 64% of TV), expiratory delay (**Te-RT**), inspiratory drive (**TV/Ti**) and expiratory drive (**TV/Te**), and non-eupneic breathing index (**NEBI**, % of breaths non-eupneic breaths including irregular events and apneas and type 1 and 2 sighs) and **NEBI/Freq** (NEBI corrected for Freq). The *Fine Pointe (BUXCO)* software constantly corrected digitized values for changes in chamber temperature and humidity. Pressure changes associated with the respiratory waveform were converted to volumes (e.g., TV, PIF, PEF) using the algorithm of Epstein and colleagues.^47,48^ Factoring in the chamber temperature and humidity, the cycle analyzers filtered the acquired signals, and algorithms (Fine Pointe, BUXCO) generated an array of box flow data that identified a waveform segment as an acceptable breath. From that data vector, the minimum and maximum values were determined. The flows at this point were “box flow” signals. From this array, the minimum and maximum box flow values were then determined and multiplied by a compensation factor provided by the selected algorithm,^47,48^ thus producing TV, PIF and PEF that are used to determine accepted and rejected waveforms. In all protocols described below, the conscious unrestrained mice were placed in the plethysmography chambers and allowed at least 60 min to acclimatize before exposure to the gas challenges.

### Protocols for gas challenges

On the day of study, C57BL/6 mice and HDAC6 KO mice were placed in whole-body plethysmography chambers and allowed at least 60 min to acclimatize and settle so that baseline breathing values could be ascertained. The mice were then exposed to a hypoxic gas (10% O_2_, 90% N_2_) challenge for 5 min after which time they were re-exposed to room air.

### Statistics

All data are shown as mean ± SEM. To determine total responses (cumulative %changes from pre-hypoxia values) during gas challenge and return to room air for each mouse, we summed the values recorded before and during the challenge and those upon return to room air. We then determined the cumulative response by the formula, total response (%change) = {[(sum of values during hypoxic challenge or return to room air) − (sum of values before hypoxic challenge)]/sum of values before hypoxic challenge} × 100. We then determined the mean and SEM of the group data. All data were analyzed by one-way or two-way ANOVA followed by Student’s modified t-test with Bonferroni corrections for multiple comparisons between means as described in detail previously.^49^

## Results

### Baseline parameters

The ages of the HDAC6 KO mice were slightly lower (−3.0%) than those of WT mice whereas the body weights of the HDAC6 KO mice were slightly higher (+ 13.2%) than the WT mice (see Table 1). As such the body weight/age ratio for the HDAC6 KO mice (0.34 ± 0.01) was higher than that of the WT mice (0.29 ± 0.1). The heavier body weights of the HDAC6 KO mice could certainly influence the findings related to flow parameters, namely, tidal volume, minute ventilation (tidal volume × frequency of breathing), peak inspiratory and expiratory flows, and EF_50_. Accordingly, (1) resting tidal volumes were lower in HDAC6 KO mice than in WT mice but were similar to one another when corrected for body weight, (2) corrections for body weights did not alter the lack of differences between the two groups with respect to minute ventilation, peak inspiratory and expiratory flows, or EF_50_. As also summarized in Table 1, resting frequency of breathing was lower in the HDAC6 KO mice than in the WT mice and inspiratory and expiratory times were longer in the HDAC6 KO mice. In addition, resting expiratory delay (Te-RT) was longer in HDAC6 KO mice than in WT mice. All other baseline parameters were similar between the two groups. It was also evident that the moment-to moment variability of many parameters was higher in the HDAC6 KO mice than in the WT mice. As shown in Table 2, values of standard deviation/corrected for mean (STDEV/mean) for frequency of breathing, minute ventilation, inspiratory time, expiratory time, end inspiratory pause and inspiratory drive were higher in the HDAC6 KO mice than in the WT mice. The finding that neither NEBI or NEBI/Freq were different between the groups suggests that the variability is due to simple changes in the breath-to-breath levels of frequency of breathing, for example, rather than enhanced expression of non-eupneic breathing including irregular breaths and apneas.

## Ventilatory responses to hypoxic gas challenges and upon return to room-air

### Frequency of breathing, tidal volume, minute ventilation

**Figure 1:**
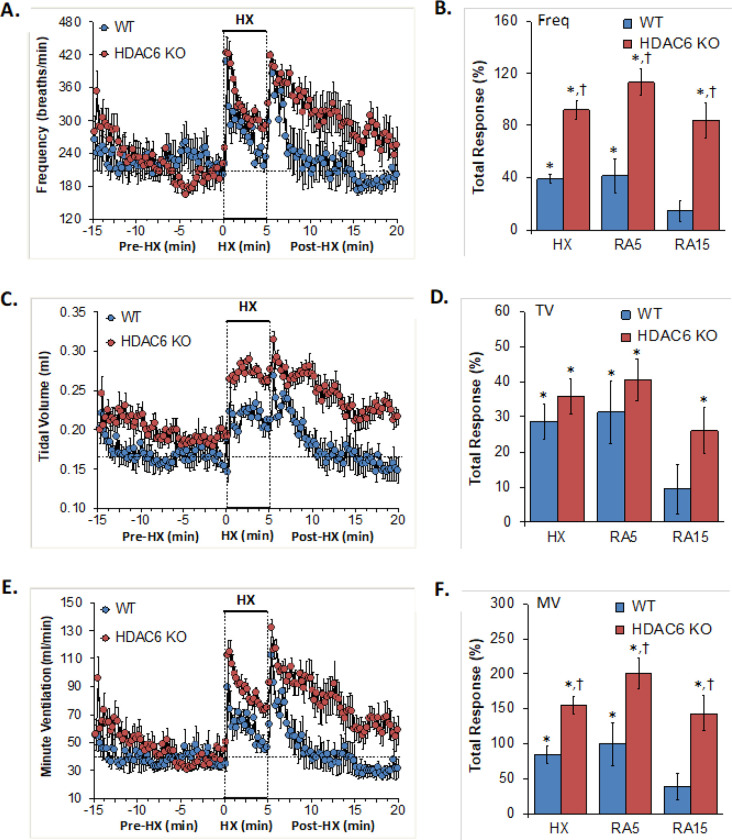
**Left-hand panels:** Frequency of breathing (Freq), tidal volume (TV) and minute ventilation (MV) before (Pre-HX), during a 5 min hypoxic gas (HX; 10% O_2_, 90% N_2_) challenge and upon return to room-air (Post-HX) in wild-type (WT) mice (n = 7) and in HDAC6 knockout (HDAC6 KO) mice (n=14). **Right-hand panels:** Total responses recorded during the hypoxic (HX) gas challenge, during the first 5 min (RA5) or 15 min (RA15) upon return to room air. All data are presented as mean ± SEM. *P < 0.05, significant response. ^†^P < 0.05, HDAC6 KO *versus* WT.

The frequency of breathing, tidal volume and minute ventilation values recorded before (Pre-HX), during a 5 min hypoxic gas (HX; 10% O_2_, 90% N_2_) challenge and upon return to room-air (Post-HX) in WT mice and in HDAC6 KO mice are shown in the left-hand panels of Fig. 1. As seen in **Panel A**, resting frequency of breathing prior to the hypoxic gas challenge was similar in WT and HDAC6 KO. The hypoxic challenge in WT mice elicited a typical increase in frequency of breathing associated with expected roll-off. The increases in Freq were somewhat higher in HDAC6 KO mice. The return to room-air elicited a typical increase in frequency of breathing in WT mice that gradually declined over the first 5 min of the recording period. The corresponding frequency of breathing values were higher in HDAC6 KO mice especially over the 5–15 min time-period when values had returned to baseline values in WT mice. The total responses summarized in **Panel B** show that the total frequency of breathing responses elicited by the hypoxic challenge (HX) and upon return to room-air (RA5 and RA15) were higher in HDAC6 KO mice than in WT mice.

As seen in **Panel C**, resting tidal volume prior to the hypoxic challenge was consistently higher in the HDAC6 KO mice than WT mice, perhaps due to the slightly larger body weights of the KO mice (see Table 1). The hypoxic challenge in the WT mice elicited a sustained increase in tidal volume that did not display roll-off. The increases in tidal volume were similarly robust in HDAC6 KO mice and reached higher values, mostly because of the higher resting values. Upon return to room-air, tidal volume values returned to pre-hypoxic levels within 5 min in WT mice but remained elevated in the HDAC6 KO mice over the 15 min recording period. The total responses summarized in **Panel D** shows that the total tidal volume responses elicited by hypoxia (HX) were similar in HDAC6 KO mice than in WT mice. The total responses upon return to room-air over the first 5 min (RA5) were similar in the HDAC6 KO and WT mice. The total tidal volume response over the entire 15 min recording period was significant in the HDAC6 KO mice but not the WT mice.

As seen in **Panel E**, resting minute ventilation prior to the hypoxic challenge was similar in WT and HDAC6 KO mice. The hypoxic challenge in the WT mice elicited a typical increase in minute ventilation that was associated with the expected roll-off. The increases in minute ventilation were consistently higher in HDAC6 KO mice. The return to room-air elicited a typical increase in minute ventilation in WT mice that subsided within 5 min. The corresponding MV values were higher in HDAC6 KO mice and remained elevated over the 15 min recording period. As seen in **Panel F**, the total minute ventilation responses elicited by hypoxia (HX) and return to room-air (RA 5 and RA15) were higher in the HDAC6 KO mice than in WT mice.

### Inspiratory time, expiratory time, expiratory time/inspiratory time

**Figure 2:**
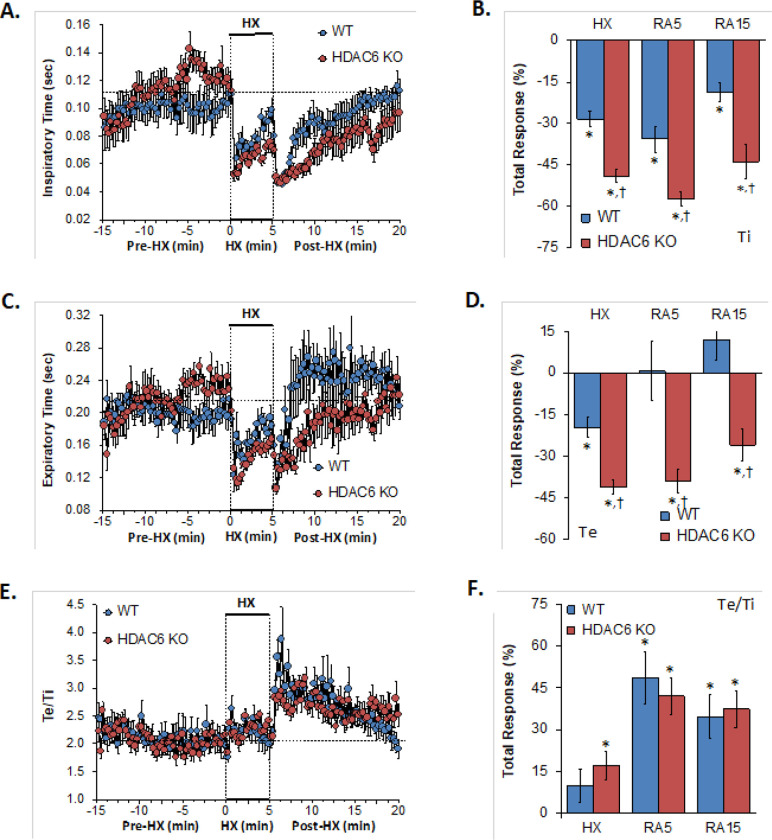
**Left-hand panels:** Inspiratory time (Ti), expiratory time (Te) and Te/Ti before (Pre-HX), during a 5 min hypoxic gas (HX; 10% O_2_, 90% N_2_) challenge and upon return to room-air (Post-HX) in wild-type (WT) mice (n = 7) and in HDAC6 knockout (HDAC6 KO) mice (n=14). **Right-hand panels:** Total responses recorded during the hypoxic (HX) gas challenge, during the first 5 min (RA5) or 15 min (RA15) upon return to room air. All data are presented as mean ± SEM. *P < 0.05, significant response. ^†^P < 0.05, HDAC6 KO *versus* WT.

The Ti, Te and Te/Ti values recorded before (Pre-HX), during a 5 min hypoxic gas (HX; 10% O_2_, 90% N_2_) challenge and upon return to room-air (Post-HX) in WT mice and in HDAC6 KO mice are shown in the left-hand panels of Fig. 2. As seen in **Panel A**, resting Ti prior to the hypoxic gas challenge tended to be higher in HDAC6 KO mice than WT mice during the 5 min period immediately before the hypoxic challenge. The hypoxic challenge in WT mice elicited a decrease in Ti that was associated with the expected roll-off. The decreases in Ti were somewhat greater in HDAC6 KO mice. The return to room-air elicited an initial further decrease in Ti in the WT mice that was followed by a gradual return toward pre-hypoxia values. Corresponding Ti values in HDAC6 KO mice showed the same pattern of changes as in WT mice but reached lower values most probably in part perhaps because of the lower values reached at the end of the hypoxic challenge. The total responses summarized in **Panel B** shows that the total Ti responses elicited by hypoxia (HX) and upon return to room-air (RA5 and RA15) were greater in HDAC6 KO mice than in WT mice.

As seen in **Panel C**, resting expiratory time prior to the hypoxic challenge tended to be higher in HDAC6 KO mice than WT mice during the 5 min period before the hypoxic challenge. The hypoxic challenge in WT mice elicited a decrease in Te that was associated with the expected roll-off. The decreases in Te were somewhat greater in HDAC6 KO mice. The return to room-air elicited an initial further decrease in Te in the WT mice that was followed by a rapid return to pre-hypoxia values. The corresponding Te values in HDAC6 KO mice followed the same pattern of changes as in WT mice but stayed at lower values for longer before returning to pre-hypoxia values. The total responses summarized in the **Panel D** shows that the total Ti responses elicited by hypoxia (HX) and upon return to room-air (RA5 and RA15) were greater in HDAC6 KO mice than in WT mice. As seen in **Panel E**, prior to the hypoxic challenge, resting Te/Ti was similar in HDAC6 KO and WT mice. The hypoxic challenge elicited minimal increases in Te/Ti in both groups. Upon return to room-air, Te/Ti rose further (with a spike evident in WT mice) before gradually declining to pre-hypoxia levels. As seen in Panel F, the the total Te/Ti responses that occurred during the hypoxic challenge (HX) and upon return to room-air (RA5 and RA15) were similar in HDAC6 KO and WT mice.

### End Inspiratory Pause, End Expiratory Pause

**Figure 3:**
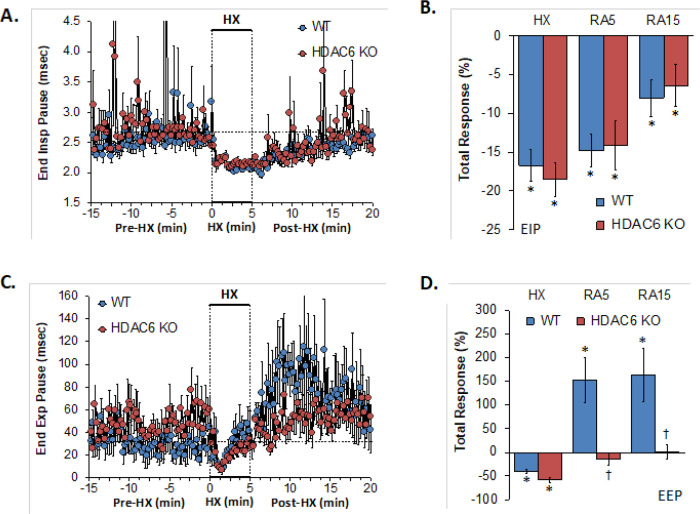
**Left-hand panels:** End inspiratory pause (EIP) and end expiratory pause (EEP) before (Pre-HX), during a 5 min hypoxic gas (HX; 10% O_2_, 90% N_2_) challenge and upon return to room-air (Post-HX) in wild-type (WT) mice (n = 7) and in HDAC6 knockout (HDAC6 KO) mice (n=14). **Right-hand panels:** Total responses recorded during the hypoxic (HX) gas challenge, during the first 5 min (RA5) or 15 min (RA15) upon return to room air. All data are presented as mean ± SEM. *P < 0.05, significant response. ^†^P < 0.05, HDAC6 KO *versus* WT.

EIP and EEP values recorded before (Pre-HX), during a 5 min hypoxic gas (HX; 10% O_2_, 90% N_2_) challenge and upon return to room-air (Post-HX) in WT mice and in HDAC6 KO mice are shown in the left-hand panels of Fig. 3. As seen in **Panel A**, resting EIP and EEP values prior to the hypoxic gas challenge were similar between HDAC6 KO mice and WT mice. The hypoxic challenge elicited similar sustained decreases in EIP in WT and HDAC6 KO mice. Upon return to room-air, the EIP values gradually returned to pre-HX levels in both groups. As seen in **Panel B**, the total EIP responses elicited by hypoxia and upon return to room-air (RA5 and RA15) were similar in HDAC6 KO and WT mice. As seen in **Panel C**, resting EEP values tended to be higher in the HDAC6 KO mice. The hypoxic challenge elicited an initial decrease in EEP in both groups of mice that tended to recover toward baseline toward the end of the challenge. Upon return to room-air, EEP rose well above baseline levels in WT mice but stayed at baseline values in the HDAC6 KO mice. As can be seen in **Panel D**, the total decreases in EEP elicited by the hypoxic challenge (HX) were similar in WT and HDAC6 KO mice. In contrast, the substantial total increases in EEP observed upon return to room-air in the WT mice (RA5 and RA15) were not seen in the HDAC6 KO mice.

### Peak Inspiratory and Expiratory Flows

**Figure 4:**
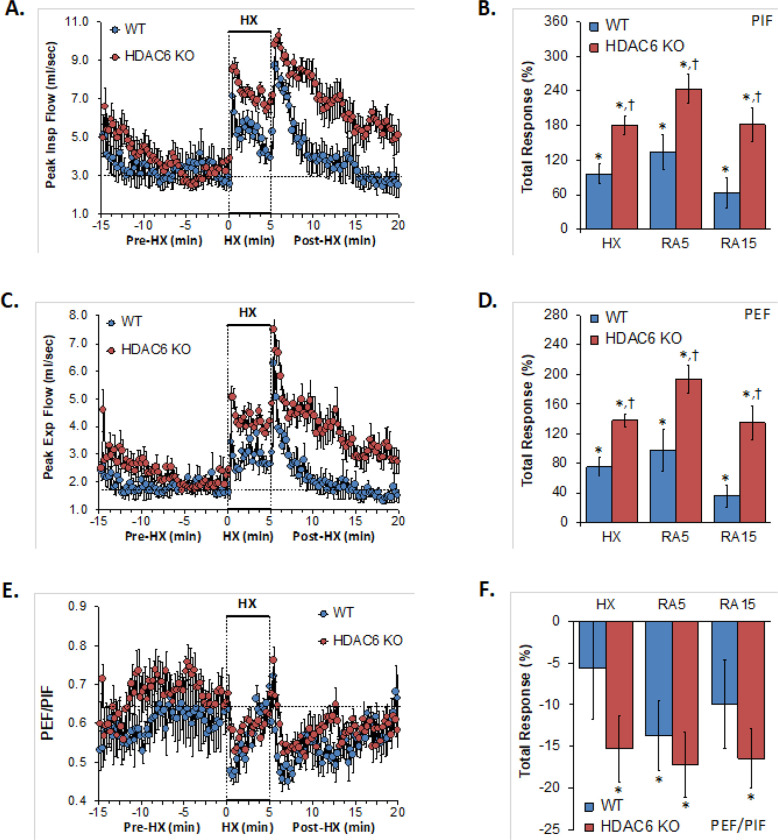
**Left-hand panels:** Peak inspiratory flow (PIF), peak expiratory flow (PEF) and PEF/PIF before (Pre-HX), during a 5 min hypoxic gas (HX; 10% O_2_, 90% N_2_) challenge and upon return to room-air (Post-HX) in wild-type (WT) mice (n = 7) and in HDAC6 knockout (HDAC6 KO) mice (n=14). **Right-hand panels:** Total responses recorded during the hypoxic (HX) gas challenge, during the first 5 min (RA5) or 15 min (RA15) upon return to room air. All data are presented as mean ± SEM. *P < 0.05, significant response. ^†^P < 0.05, HDAC6 KO *versus* WT.

The PIF and PEF values recorded before (Pre-HX), during a 5 min hypoxic gas (HX; 10% O_2_, 90% N_2_) challenge and upon return to room-air (Post-HX) in WT mice and in HDAC6 KO mice are shown in the left-hand panels of Fig. 4. As seen in **Panels A and C**, resting PIF and PEF values were similar between the HDAC6 KO mice and WT mice. The HX challenge elicited sustained increases in PIF and PEF in WT mice. These responses were markedly augmented in HDAC6 KO mice. Upon return to room-air, PIF and PEF spiked upward before gradually returning to baseline levels in WT and HDAC6 KO mice with PIF and PEF values remaining substantially higher in HDAC6 KO mice. As seen in **Panels B and D**, total PIF and PEF responses elicited by hypoxia (HX) and upon return to room-air (RA5 and RA15) were greater in the HDAC6 KO than in the WT mice.

### EF_50_, relaxation time, expiratory delay (Te-RT)

**Figure 5:**
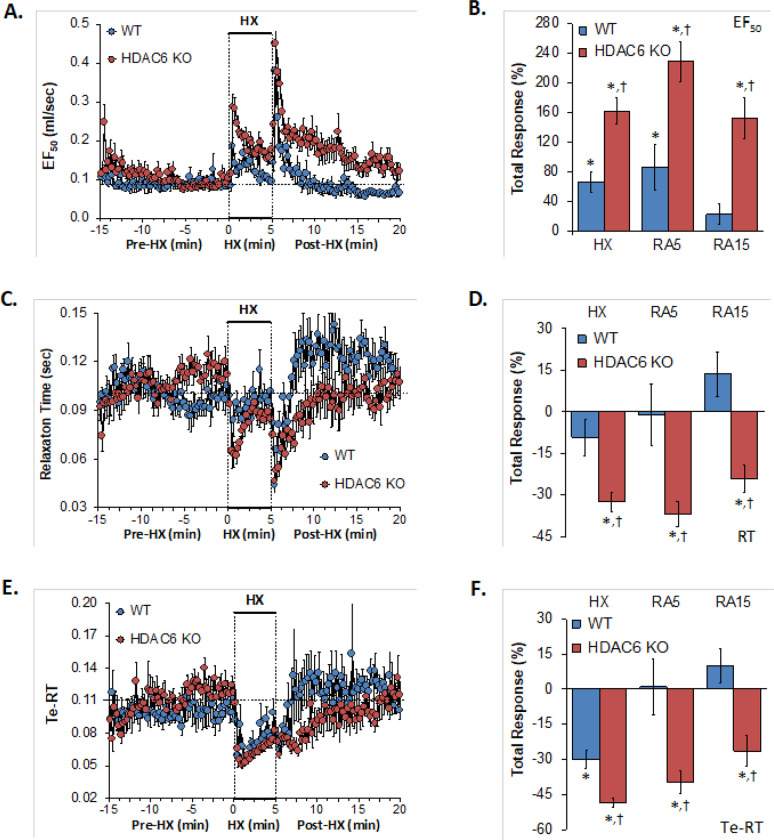
**Left-hand panels:** Expiratory flow at 50% tidal volume (EF_50_), relaxation time (RT) and expiratory delay (Te-RT) before (Pre-HX), during a 5 min hypoxic gas (HX; 10% O_2_, 90% N_2_) challenge and upon return to room-air (Post-HX) in wild-type (WT) mice (n = 7) and in HDAC6 knockout (HDAC6 KO) mice (n=14). **Right-hand panels:** Total responses recorded during the hypoxic (HX) gas challenge, during the first 5 min (RA5) or 15 min (RA15) upon return to room air. All data are presented as mean ± SEM. *P < 0.05, significant response. ^†^P < 0.05, HDAC6 KO *versus* WT.

EF_50_, relaxation time, expiratory delay (Te-RT) values recorded before (Pre-HX), during a 5 min hypoxic gas (HX; 10% O_2_, 90% N_2_) challenge and upon return to room-air (Post-HX) in WT mice and in HDAC6 KO mice are shown in the left-hand panels of Fig. 5. As seen in **Panel A**, resting EF_50_ values were similar between the HDAC6 KO mice and WT mice. The HX challenge elicited greater increases in EF_50_ in the HDAC6 KO mice than in the WT mice. These responses were markedly augmented in HDAC6 KO mice. The responses that occurred upon return to room-air were also markedly greater in the HDAC6 KO mice. As seen in **Panel B**, the total increases EF_50_ elicited by the hypoxic challenge and upon return to room-air (RA5 and RA15) were greater in the HDAC6 KO than in the WT mice. As seen in **Panel C**, resting relaxation time values were similar in HDAC6 KO mice and WT mice. The hypoxic challenge elicited minimal changes in relaxation time in the WT mice but substantial initial falls in the HDAC6 KO mice that recovered within 3 min of the hypoxic challenge. Relaxation time dropped substantially upon return to room-air in the WT and HDAC6 KO mice. Relaxation time then rose above baseline values in the WT mice but returned to baseline values in HDAC6 KO mice. As seen in **Panel D**, total decreases in relaxation elicited by hypoxia and upon return to room-air (RA5 and RA15) were greater in HDAC6 KO than in WT mice. As seen in **Panel E**, resting expiratory delay (Te-RT) values tended to be higher in HDAC6 KO mice than in the WT mice immediately before the hypoxic challenge. The hypoxic challenge elicited slightly greater decreases in expiratory delay in HDAC6 KO mice. Expiratory delay returned rapidly to baseline levels in the WT mice whereas these values remained below baseline values in HDAC6 KO mice for 5–6 min. As seen in **Panel F**, the total decreases in expiratory delay elicited during hypoxic challenge (HX) were greater in HDAC6 KO than in WT mice. Decreases in expiratory delay upon return to room air (RA5 and RA15) occurred in the HDAC6 KO mice only.

### Inspiratory and Expiratory Drives

**Figure 6:**
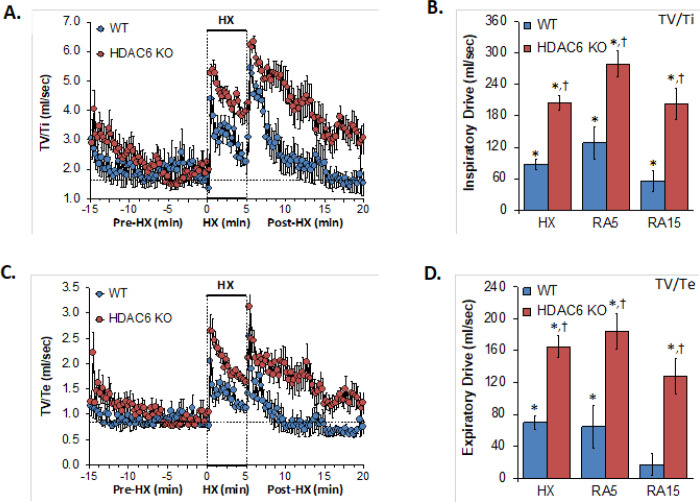
**Left-hand panels:** Inspiratory drive (tidal volume/inspiratory time, TV/Ti) and expiratory time (tidal volume/expiratory time, TV/Te) before (Pre-HX), during a 5 min hypoxic gas (HX; 10% O_2_, 90% N_2_) challenge and upon return to room-air (Post-HX) in wild-type (WT) mice (n = 7) and in HDAC6 knockout (HDAC6 KO) mice (n=14). **Right-hand panels:** Total responses recorded during the hypoxic (HX) gas challenge, during the first 5 min (RA5) or 15 min (RA15) upon return to room air. All data are presented as mean ± SEM. *P < 0.05, significant response. ^†^P < 0.05, HDAC6 KO *versus* WT.

Inspiratory and expiratory drive values recorded before (Pre-HX), during a 5 min hypoxic gas (HX; 10% O_2_, 90% N_2_) challenge and upon return to room-air (Post-HX) in WT mice and in HDAC6 KO mice are shown in the left-hand panels of Fig. 6. As seen in **Panels A and C**, resting drives prior to the hypoxic challenge were similar in HDAC6 KO mice and WT mice. The hypoxic challenge elicited sustained increases in inspiratory drive and expiratory drive in WT mice. These responses were markedly augmented in HDAC6 KO mice. Upon return to room-air, the inspiratory drive and expiratory drive values spiked upward in WT mice before gradually returning to baseline levels. These values were considerable higher in the HDAC 6 KO mice. As seen in **Panels B and D**, the responses elicited during the hypoxic challenge (HX) and upon return to room-air (RA5 and RA15) were greater in HDAC6 KO mice than WT mice.

### NEBI, NEBI/Freq

**Figure 7:**
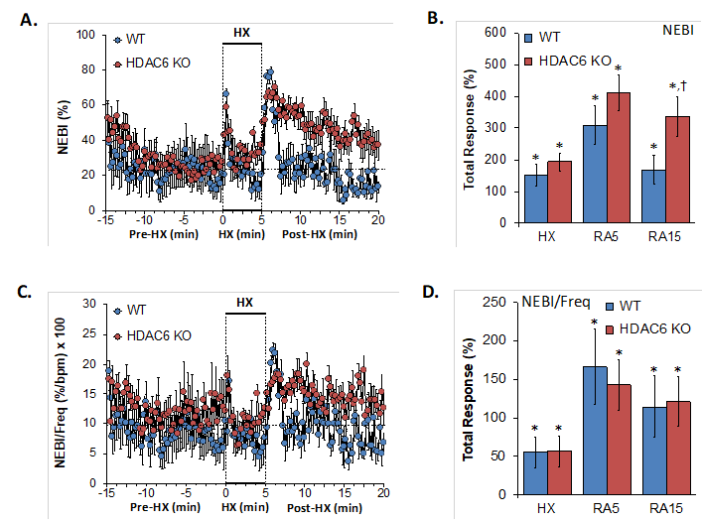
**Left-hand panels:** Non-eupneic breathing index (NEBI) and NEBI/frequency of breathing (NEBI/Freq) before (Pre-HX), during a 5 min hypoxic gas (HX; 10% O_2_, 90% N_2_) challenge and upon return to room-air (Post-HX) in wild-type (WT) mice (n = 7) and in HDAC6 knockout (HDAC6 KO) mice (n=14). **Right-hand panels:** Total responses recorded during the hypoxic (HX) gas challenge, during the first 5 min (RA5) or 15 min (RA15) upon return to room air. All data are presented as mean ± SEM. *P < 0.05, significant response. ^†^P < 0.05, HDAC6 KO *versus* WT.

Non-eupneic breathing index (NEBI) and NEBI/Freq values recorded before (Pre-HX), during a 5 min hypoxic gas (HX; 10% O_2_, 90% N_2_) challenge and upon return to room-air (Post-HX) in WT mice and in HDAC6 KO mice are shown in the left-hand panels of Fig. 7. As seen in **Panel A**, resting NEBI values prior to the hypoxic challenge were similar in HDAC6 KO mice and WT mice. The hypoxic challenge elicited similar increases in NEBI in WT and HDAC6 KO mice. The return to room-air caused remarkable increases in NEBI in both groups with NEBI subsiding more rapidly in the WT mice. These values were considerable higher in the HDAC 6 KO mice. As seen in **Panel B**, the increases in NEBI elicited during the hypoxic challenge (HX) and during the first 5 min upon return to room-air (RA5) were similar in the WT and HDAC6 KO mice whereas the overall increase in NEBY (RA15) was much greater in the HDAC6 mice. and RA15) were greater in HDAC6 KO mice than WT mice. As seen in **Panels C and D**, normalizing the changes in NEBI for the changes in frequency of breathing (NEBI/Freq) resulted in the changes during hypoxia (HX) and upon return to room-air (RA5 and RA15) that were similar in both groups of mice.

### Body weight considerations

The heavier body weights of HDAC 6 KO mice may influence findings related to the effects of hypoxic challenge and return to room-air on flow parameters; tidal volume, minute ventilation, peak inspiratory and expiratory flows, and EF_50_. Table 3 summarizes the total arithmetic changes in ventilatory parameters during hypoxic gas challenge with flow parameters also shown corrected for body weight. The total arithmetic changes for frequency of breathing, inspiratory and expiratory times, expiratory time/expiratory time, end inspiratory and expiratory pauses, relaxation time, expiratory time-relaxation time, NEBI and NEBI/Freq provide the exact same conclusions given by the %change data provided in Figs. 1–7 in that hypoxia-mediated changes in these parameters were greater in the HDAC6 KO mice. Second, the delta changes in flow variables corrected for body weights (delta/body weight) for tidal volume, minute ventilation, peak inspiratory and expiratory flows, EF_50_ and inspiratory and expiratory drives were also consistent with %change data provided in Figs. 1–7 in that except for tidal volume, the hypoxia-mediated changes in these parameters were greater in the HDAC6 KO mice than in the WT mice.

## Discussion

The C57BL/6 mouse is a common inbred strain that is widely used in ventilatory and pulmonary function studies^50,51^ and to produce mice lacking genes for numerous functional proteins.^41,52–54^ The C57BL/6 mouse is used as a mouse model with “normal” physiology and indeed they display many “normal” traits.^41–44,55–58^ For example, resting systemic and pulmonary arterial blood pressures at rest and cardiovascular responses upon challenges with hypoxic, hypercapnic and hypoxic-hypercapnic gas mixtures are representative of other healthy mouse and rat strains.^56–58^ Moreover, the ability of hypercapnia to modulate the effects of hypoxia on arterial blood pressure (hypoxia elicits pronounced depressor response, hypercapnia elicits a minor pressor response, hypoxia-hypercapnia elicits a minimal response) is to be intact in C57BL/6 mice.^56,57^ As such, C57BL/6 mice have been used extensively to study the effects of hypoxic, hypercapnic and hypoxic-hypercapnic gas challenges on ventilatory function^41–44^ and disordered breathing during both wakefulness and sleep.^59–67^ Despite being slightly younger, the HDAC6 KO mice were slightly heavier than their WT (C57BL/6) littermate controls. Whether this means that deletion of HDAC6 affects body metabolism or other factors regulating general health/body weight in C56BL/6 mice are yet to be established. In addition, the loss of HDAC6 could directly/indirectly impact ventilatory parameters in C57BL/6 mice by numerous mechanisms. For example, HDAC6 exists in smooth muscle and vascular endothelium of pulmonary arteries and Inhibition of HDAC6 improves the functions of both cell types.^68,69^

### Resting ventilatory parameters

A key finding of this study was that baseline (pre-HX gas challenge) frequency of breathing values were lower in the HDAC6 KO mice than in the WT mice. The reduced frequency of breathing in HDAC6 KO mice was accompanied by longer inspiratory and expiratory times. These findings certainly suggest that the possible presence of HDAC6 in key brainstem sites controlling respiratory frequency such as the NTS has a vital role in setting resting inspiratory and expiratory times. Moreover, the increased baseline variability in breathing parameters in HDAC6 KO mice (in the absence of enhanced non-eupneic breathing) certainly suggests that the presence of HDAC6 is essential for normal patterning of breathing. Similarly, the findings that end expiratory pause and expiratory delay (Te-RT) were greater in the HDAC6 KO mice suggests that HDAC6 is important for regulation of expiratory dynamics. Finally, the findings that the majority of the resting ventilatory parameters were similar in HDAC6 KO and WT mice (e.g., tidal volume, peak inspiratory and expiratory flows) does not negate a role for HDAC6 in the control of these parameters but rather that C57BL/6 mice are able to compensate for the loss of this important signaling element.

### Ventilatory Responses to Hypoxic Gas Challenge

The hypoxic gas challenge elicited substantially greater increases in frequency of breathing (but not tidal volume) and therefore minute ventilation in HDAC6 KO mice than in WT mice. These novel findings suggest that stabilizing microtubules has a very important positive effect on respiratory timing but perhaps not ventilatory mechanics. The carotid body and chemoafferents in the carotid sinus nerve play an essential role in detecting and transmitting hypoxic signals to the commissural nuclei tractus solitarii in the brainstem^26–29^ and we have provided evidence that hypoxic ventilatory responses are markedly reduced in freely-moving male C57BL/6 (WT) mice with bilateral carotid sinus nerve transection.^43^ Although currently lacking, evidence that HDAC6 exists within the carotid bodies and key brain structures such as the nucleus tractus solitarii (see below for further discussion) would supports our evidence that HDAC6 is vital to the robustness of hypoxic ventilatory signaling. The hypoxia-induced increases in frequency of breathing were, as expected, associated with temporally consistent decreases in inspiratory and expiratory times in WT and HDAC6 KO mice. The decreases in inspiratory and expiratory times and were greater than the HDAC6 KO mice than the WT mice, consistent with the more pronounced increases in Freq in HDAC6 KO mice. The decrease in inspiratory time was somewhat greater than the decrease in Te in the WT and HDAC6 KO mice such that there was a slight increase in expiratory quotient (Te/Ti ratio) in both groups of mice with the increase in this ratio being larger in the HDAC6 KO mice. The combinations of increased tidal volume coupled to decreases in inspiratory and expiratory times resulted in marked increases in inspiratory drive (TV/Ti) and expiratory drive (TV/Te) in both groups but which were substantially larger in the HDAC6 KO mice. Again, although data is not available as to the precise brain sites that HDAC6 may participate in hypoxic signaling, it is known that HDAC6 exists widely throughout the brain although it is particularly associated with serotonergic neurons such as the dorsal and median raphe nuclei^70–72^ that are known to have important roles in the control of ventilatory processes^73–75^ although it appears that whereas these neurons play a key role in the expression of the ventilatory responses to hypercapnic challenges, they do not play a major role in expression of ventilatory responses to hypoxic gas challenges.^76–82^

As would be expected, end inspiratory and expiratory pauses decreased during exposure to the hypoxic challenge in the WT and HDAC6 KO mice. The decreases in end inspiratory pause were identical in the WT and HDAC6 KO mice whereas the decreases in end inspiratory pause were substantially greater in the HDAC6 KO mice. In addition, although relaxation times and expiratory delay (expiratory time − relaxation time) shortened remarkably during the hypoxic challenge in both groups, the decreases were substantially greater in the HDAC6 KO mice. Moreover, the increases in PIF, PEF and EF_50_ during the hypoxic challenge were dramatically augmented in the HDAC6 KO mice compared to the WT mice. Again, although it is not known if HDAC6 exists in the diaphragm and/or chest wall, histone deacetylases do exist in skeletal muscle,^83–85^ and as such reduced expression and/or pharmacological blockade of HDAC6 may increase the force of contraction generated by ventilatory muscles thereby enhancing PIF, PEF and EF_50_ responses during hypoxic gas challenges. Finally, the hypoxic challenge caused a substantially greater increase in the non-eupneic index (NEBI; e.g., disordered breathing, apneas, type 1 and 2 sighs) of HDAC6 KO mice than in WT mice although when corrected for the values for frequency of breathing (also more greatly elevated in HDAC6 KO mice) NEBI/Freq was similar in both groups. Although, we have argued that NEBI may reach higher values with higher values of frequency of breathing,^44–46^ this may not always be the case and so it remains plausible that the lack of HDAC6 destabilizes breathing patterns during hypoxic challenges.

### Ventilatory Responses upon Return to Room-Air

The return to room-air in mice having undergone hypoxic gas challenges often results in an abrupt dramatic increase in Freq, TV and therefore MV in mice^40–46, 86^ that can result in unstable breathing.^86–89^ The mechanisms responsible for post-hypoxia alterations in breathing have received considerable investigation and at present, evidence favors disturbances in central signaling^87,90^ including the pons^91,92^ rather than processes within the carotid bodies.^93,94^ The present study demonstrated that C57BL/6 WT mice displayed the expected abrupt increase frequency of breathing, tidal volume and minute ventilation upon return to room-air which returned to baseline within 5 min. The increases in frequency of breathing and minute ventilation (but not tidal volume) upon return to room-air were greater in the HDAC6 KO mice than WT mice over the first 5 min of return to room-air and took substantially longer to return to baseline values. These results clearly suggest that HDAC6 within peripheral and central neural structures normally plays a vital role in the ventilatory adaptations that occur upon the return to room-air. As would be expected, the decreases in inspiratory and expiratory times were greater in the HDAC6 KO mice than the WT mice over the first 5 min following return to room-air and were sustained for a longer period of time. A careful view of the data shows that expiratory time returned to pre-hypoxia (baseline) values relatively abruptly in the WT mice whereas it remained decreased for 5–10 min in HDAC6 KO mice. This evidence is strongly supported by the finding that the abrupt and sustained increases in end expiratory pause that occurred upon return to room-air in WT mice was virtually absent in the HDAC6 KO mice. This contrasts with the gradual return of end inspiratory pause to baseline levels upon return to room-air in the WT and HDAC6 KO mice. Taken together, this data provides evidence that HDAC6 has a major role in brain neural circuitry regulating expiratory timing. The increases in peak inspiratory and expiratory flows and EF_50_ upon return to room-air were greater over the first 5 min in HDAC6 KO mice than in WT mice and remained greater for longer periods. Again, enhanced activity of skeletal muscle in chest wall and diaphragm may be directly responsible for the enhanced responses in the HDAC6 KO mice although augmented central output to these muscles cannot be discounted. The findings that the decreases in relaxation time and expiratory delay (Te-RT) were remarkably greater in the HDAC6 KO mice also points to an important role for HDAC6 in expiratory control processes. Taking the changes in tidal volume and inspiratory and expiratory times into account, it was evident that the increases in inspiratory drive (TV/Ti) and expiratory drive (TV/Te) upon return to room-air were substantially greater in HDAC6 KO mice. Taken together, the data reinforce the overall impression that HDAC6 has a major role in regulating inspiratory and expiratory timing in C57BL/6 mice. The finding that the increase in NEBI upon return to room-air was greater in the HDAC6 KO mice tentatively suggests that HDAC6 plays a vital role in ventilatory stability during this phase and that the loss of HDAC6 may contribute to ventilatory instability (increased expression of abnormal breaths and apneas) during this phase and perhaps in general. The finding that post-hypoxia (post-apnea) breathing in humans is associated with severe glottal closures^95,96^ raises the possibility that decreased expression of HDAC6 may contribute to obstruction of the upper airway in patients with obstructive apneas and perhaps the expression of central apneas.

### Summary

The genetic bases for different breathing patterns in mice at rest and in response to hypoxic and hypercapnic challenges in mouse strains have received extensive investigation^96–105^ as have neurochemical processes,^66,67,88,89,106–108^ and structural features of respiratory structures such as the carotid bodies.^109–111^ The possibility that HDAC6 is a key player in the genetic factors that regulate breathing opens up intriguing avenues of research and especially testing whether selective HDAC6 inhibitors such as CAY10603, Tubacin and Nexturastat^112–116^ augment and/or stabilize ventilatory responses to hypoxic and/or hypercapnic gas challenges in mouse models such as C57BL/6 mice.^63–65,^ 88,89 The results of the present study in male mice raises the question of whether female HDAC6 KO mice will also display many of the ventilatory features displayed by males and especially the ventilatory responses to hypoxia and those that occur upon return to room-air. The question of how male and female HDAC6 KO mice respond to a hypercapnic gas challenge is also of great interest with respect to understanding the physiological role of HDAC6.

## Figures and Tables

**Table 1:** Baseline parameters in wild-type (WT) and HDAC6 knockout (HDAC6 KO) mice with the shown delta/body weight values represent the actual values multiplied by 1,000.

Parameter	Abbreviation	WT mice	HDAC6 KO mice
Number of mice in each group		7	14
Age, (days)		94.7 ± 0.9	91.9 ± 0.6*
Body Weight, (g)		27.3 ± 0.6	30.9 ± 0.9*
Body Weight/Age, (g/days)		0.29 ± 0.1	0.34 ± 0.01*
Frequency, (breaths/min)	Freq	200 ± 6	170 ± 5*
Inspiratory Time, (sec)	Ti	0.111 ± 0.003	0.133 ± 0.005*
Expiratory Time, (sec)	Te	0.210 ± 0.008	0.253 ± 0.009*
Expiratory/Inspiratory Time	Te/Ti	2.01 ± 0.08	1.97 ± 0.10
End Inspiratory Pause, (msec)	EIP	2.59 ± 0.09	2.68 ± 0.07
End Expiratory Pause, (msec)	EEP	30.6 ± 6.1	61.0 ± 8.5*
Tidal Volume, (ml)	TV	0.166 ± 0.010	0.200 ± 0.008*
*TV/body weight, (ml/g) × 1000	TV/BW	6.10 ± 0.35	6.51 ± 0.26
Minute Ventilation, (ml/min)	MV	32.9 ± 3.0	34.9 ± 2.5
*MV/body weight, (ml/g) × 1000	MV/BW	1215 ± 133	1134 ± 79
Peak Inspiratory Flow, (ml/sec)	PIF	2.73 ± 0.38	2.72 ± 0.26
*PIF/body weight, (ml/g) × 1000	PIF/BW	101.4 ± 16.4	88.5 ± 8.5
Peak Expiratory Flow (ml/sec)	PEF	1.63 ± 0.15	1.77 ± 0.10
*PEF/body weight, (ml/g) × 1000	PEF/BW	60.3 ± 6.2	57.6 ± 3.3
PEF/PIF		0.62 ± 0.04	0.71 ± 0.03
*(PEF/PIF)/body weight (ratio/g) × 1000		22.7 ± 1.1	23. 2 ± 1.1
Air-flow at 50% expired TV (ml/sec)	EF_50_	0.079 ± 0.007	0.077 ± 0.006
*EF_50_/body weight (ratio/g) × 1000	EF_50_/BW	2.93 ± 0.30	2.51 ± 0.21
Relaxation Time	RT	0.104 ± 0.006	0.122 ± 0.005
Expiratory Delay	Te-RT	0.106 ± 0.005	0.128 ± 0.005*
Inspiratory Drive (ml/sec)	TV/Ti (InspD)	1.62 ± 0.15	1.46 ± 0.10
*(TV/Ti)/body weight (ratio/g) × 1000	InspD/BW	59.7 ± 6.4	47.5 ± 2.9
Expiratory Drive (ml/sec)	TV/Te (ExpD)	0.80 ± 0.05	0.75 ± 0.03
*(TV/Te)/body weight (ratio/g) × 1000	ExD/BW	29.5 ± 2.3	24.5 ± 1.1
Non-eupneic breathing Index (%)	NEBI	10.3 ± 1.3	13.8 ± 1.8
NEBI/Freq (%/(breaths/min))	NEBI/Freq	0.057 ± 0.012	0.088 ± 0.013

The data are presented as mean ± SEM.

* *P* < 0.05, histone deacetylase 6 knockout (HDAC6 KO) mice *versus* wild-type (WT).

**Table 2:** Variability in resting variables in wild-type (WT) and HDAC6 knockout (HDAC6 KO) mice

Parameter	Parameter	WT mice	HDAC6 KO mice
**Frequency (breaths/min)**	STDEV	45.5 ± 7.4	74.7 ± 7.8*
	Mean	228 ± 20	225 ± 16
	STDEV/Mean	0.195 ± 0.019	0.325 ± 0.023*
**Tidal Volume (ml)**	STDEV	0.031 ± 0.003	0.038 ± 0.003
	Mean	0.170 ± 0.010	0.200 ± 0.008
	STDEV/Mean	0.180 ± 0.014	0.188 ± 0.009
**Minute Ventilation (ml/min)**	STDEV	12.5 ± 2.6	22.8 ± 3.0*
	Mean	39.4 ± 5.7	47.1 ± 5.1
	STDEV/Mean	0.304 ± 0.028	0.474 ± 0.036*
**Inspiratory Time (sec)**	STDEV	0.017 ± 0.002	0.032 ± 0.003*
	Mean	0.100 ± 0.009	0.113 ± 0.007
	STDEV/Mean	0.179 ± 0.030	0.297 ± 0.032*
**Expiratory Time (sec)**	STDEV	0.037 ± 0.004	0.059 ± 0.003*
	Mean	0.200 ± 0.010	0.217 ± 0.010
	STDEV/Mean	0.184 ± 0.020	0.277 ± 0.019*
**Expiratory Time/Inspiratory Time**	STDEV	0.457 ± 0.044	0.580 ± 0.028*
	Mean	2.122 ± 0.135	2.076 ± 0.082
	STDEV/Mean	0.218 ± 0.024	0.286 ± 0.019
**End Inspiratory Pause (msec)**	STDEV	0.440 ± 0.16	1.56 ± 0.68*
	Mean	2.58 ± 0.10	2.84 ± 0.14
	STDEV/Mean	0.165 ± 0.054	0.460 ± 0.149*
**End Expiratory Pause (msec)**	STDEV	29.7 ± 2.3	44.4 ± 4.4*
	Mean	31.0 ± 6.0	47.9 ± 4.9*
	STDEV/Mean	1.156 ± 0.209	0.954 ± 0.058
**Peak Inspiratory Flow (PIF, ml/sec)**	STDEV	1.215 ± 0.227	1.895 ± 0.168
	Mean	3.36 ± 0.59	3.91 ± 0.46
	STDEV/Mean	0.363 ± 0.035	0.508 ± 0.032*
**Peak Expiratory Flow (PEF, ml/sec)**	STDEV	0.575 ± 0.099	1.030 ± 0.130*
	Mean	1.85 ± 0.22	2.35 ± 0.19*
	STDEV/Mean	0.301 ± 0.025	0.426 ± 0.032*
**PEF/PIF**	STDEV	0.109 ± 0.016	0.133 ± 0.008
	Mean	0.529 ± 0.027	0.670 ± 0.032*
	STDEV/Mean	0.180 ± 0.020	0.200 ± 0.010
**EF_50_(ml/sec)**	STDEV	0.027 ± 006	0.054 ± 0.008*
	Mean	0.089 ± 0.010	0.106 ± 0.010
	STDEV/Mean	0.298 ± 0.0.051	0.489 ± 0.047*
**Relaxation Time (sec)**	STDEV	0.019 ± 0.002	0.027 ± 0.001
	Mean	0.100 ± 0.004	0.106 ± 0.005
	STDEV/Mean	0.191 ± 0.024	0.259 ± 0.016
**Expiratory Time − Relaxation Time**	STDEV	0.024 ± 0.002	0.037 ± 0.002*
	Mean	0.100 ± 0.007	0.111 ± 0.005
	STDEV/Mean	0.246 ± 0.019	0.342 ± 0.021*
**Inspiratory Drive (ml/sec)**	STDEV	0.728 ± 0.145	1.140 ± 0.118*
	Mean	1.98 ± 0.34	2.28 ± 0.27
	STDEV/Mean	0.363 ± 0.036	0.517 ± 0.036*
**Expiratory Drive (ml/sec)**	STDEV	0.251 ± 0.051	0.486 ± 0.076*
	Mean	0.90 ± 0.01	1.08 ± 0.10
	STDEV/Mean	0.264 ± 0.028	0.428 ± 0.041
**NEBI (%)**	STDEV	18.9 ± 2.5	20.9 ± 1.0
	Mean	24.0 ± 8.0	30.3 ± 5.5
	STDEV/Mean	1.099 ± 0.160	1.031 ± 0.157
**NEBI/Freq (%/(breaths/min))**	STDEV	0.068 ± 0.004	0.088 ± 0.007
	Mean	0.089 ± 0.022	0.122 ± 0.020
	STDEV/Mean	0.999 ± 0.163	0.953 ± 0.131

The data are presented as mean ± SEM.

* *P* < 0.05, histone deacetylase 6 knockout (HDAC6 KO) mice *versus* wild-type (WT).

**Table 3:** Total arithmetic responses during hypoxic gas challenge

Parameter	Parameter	WT mice	HDAC6 KO mice
**Flow-*independent* parameters**			
Frequency (breaths/min)	Delta response	+1570 ± 157*	+ 3076 ± 212*,^†^
Inspiratory Time (sec)	Delta response	−0.63 ± 0.07*	−1.33 ± 0.09*,^†^
Expiratory Time (sec)	Delta response	−0.85 ± 0.16*	−2.12 ± 0.19*,^†^
Expiratory Time/Inspiratory Time	Delta response	+ 3.72 ± 2.27	+ 5.66 ± 1.82*
End Inspiratory Pause (msec)	Delta response	−8.84 ± 1.44*	−10.28 ± 1.41*
End Expiratory Pause (msec)	Delta response	−227 ± 54*	−781 ± 153*,^†^
Relaxation Time (sec)	Delta response	−0.22 ± 0.13	−0.82 ± 0.11*,^†^
Expiratory Time − Relaxation Time	Delta response	−0.63 ± 0.07*	−1.26 ± 0.08*,^†^
NEBI (%)	Delta response	+ 292 ± 55*	+ 420 ± 40*,^†^
NEBI/Freq (%/(breaths/min))	Delta response	+ 0.46 ± 0.17*	+ 0.40 ± 0.24*
**Flow-dependentparameters**			
Tidal Volume (ml)	Delta response	+ 0.91 ± 0.14*	+ 1.34 ± 0.15*
	Delta/Body Weight	+ 0.033 ± 0.005*	+ 0.045 ± 0.006
Minute Ventilation (ml/min)	Delta response	+ 531 ± 74*	+ 1024 ± 54*,^†^
	Delta/Body Weight	+ 19.3 ± 2.5*	+ 33.6 ± 2.2*,^†^
PIF (ml/sec)	Delta response	+ 46.1 ± 6.6*	+ 88.5 ± 4.3*,^†^
	Delta/Body Weight	+ 1.67 ± 0.22*	+ 2.90 ± 0.17*,^†^
PEF (ml/sec)	Delta response	+ 23.6 ± 3.4*	+ 47.7 ± 2.7*,^†^
	Delta/Body Weight	+ 0.863 ± 0.12*	+ 1.55 ± 0.09*,^†^
PEF/PIF	Delta response	− 0.93 ± 0.79	− 2.39 ± 0.51
	Delta/Body Weight	− 0.032 ± 0.029	− 0.081 ± 0.016
**Flow-*independent* parameters**			
EF50 (ml/sec)	Delta response	+ 0.98 ± 0.18*	+ 2.34 ± 0.16*,^†^
	Delta/Body Weight	0.035 ± 0.006*	+ 0 076 ± 0 006*,^†^
Inspiratory Drive (ml/sec	Delta response	+ 26.9 ± 2.4*	+ 57.5 ± 3.0*,^†^
	Delta/Body Weight	+ 0.99 ± 0.08*	+ 1.88 ± 0.11 *,^†^
Expiratory Drive (ml/sec)	Delta response	+ 11.1 ± 1.6*	24.3 ± 17*,^†^
	Delta/Body Weight	+ 0.40 ± 0.05*	+ 0.80 ± 0.06*,^†^

WT, wild-type; HDAC6 KO histone deacetylase 6 knockout mice; NEBI, non-eupneic breathing index; Freq, frequency of breathing; PIF, peak inspiratory flow; PEF, peak expiratory flow; EF50, airflow at 50% expired tidal volume. The data are presented as mean ± SEM.

* *P* < 0.05, histone deacetylase 6 knockout (HDAC6 KO) mice *versus* wild-type (WT).

## Data Availability

The datasets used and/or analyzed during the current study available from the corresponding author on reasonable request.
